# Pathogenicity Evaluation and Virulence Gene Identification of an Attenuated Duck Enteritis Virus

**DOI:** 10.3390/microorganisms13112537

**Published:** 2025-11-05

**Authors:** Xiaona Shi, Haibin Zhuang, Dun Shuo, Luzhao Li, Shenghui Pan, Zihua Wu, Mei Tang, Wenxia Yang, Qinfang Liu, Chunxiu Yuan, Dawei Yan, Xue Pan, Bangfeng Xu, Zhifei Zhang, Minghao Yan, Qiaoyang Teng, Zejun Li

**Affiliations:** Shanghai Veterinary Research Institute, Chinese Academy of Agricultural Sciences, Shanghai 200241, China; shixiaonare@163.com (X.S.); zhuanghaibin20@163.com (H.Z.); dun.shuo@hotmail.com (D.S.); luzhao.li@wur.nl (L.L.); panshenghui37@163.com (S.P.); wuzihua6@163.com (Z.W.); 18773546303@163.com (M.T.); yangwx2024@163.com (W.Y.); liuqinfang@shvri.ac.cn (Q.L.); yuanchx@shvri.ac.cn (C.Y.); yandawei@shvri.ac.cn (D.Y.); panxue@shvri.ac.cn (X.P.); xubangfeng@163.com (B.X.); nzhangzhifei@163.com (Z.Z.);

**Keywords:** duck enteritis virus, pathogenicity, attenuation, vaccine

## Abstract

Duck enteritis virus (DEV), an epornitic pathogen, causes substantial economic losses in the commercial duck industry and poses persistent risks to wild and migratory waterfowl populations. However, due to the large genomic capacity of the DEV, the understanding of the virulence-associated genes of DEV is still limited. In previous studies, we developed an attenuated strain E74 by serial passage of a virulent strain E1 on primary chicken embryo fibroblasts (CEFs). The bird experiment showed that the mortality rate of E1 on ducks reached 100%, and high-titered viruses were detected in all tested tissue samples. In contrast, the E74 virus has lost its pathogenicity in ducks and can only be detected at a relatively low viral load in the spleen. Furthermore, the E74 stimulated a significant increase in antibodies in the ducks at 7 days post-inoculation. To further investigate the molecular basis of the attenuation of DEV in ducks, the complete genomes of E74 and E1 were sequenced and analyzed. Compared with E1, E74 had a 5152 bp deletion in the UL region, which resulted in the lack of the hypothetical protein, LORF5, UL55 and LORF4 genes. To test the influence of the deletion on the viral pathogenicity, a rescued virus rE1-Δ5152 with the 5152 bp deletion in the UL region was generated on the E1 backbone. Animal experiments showed that the lethality of rE1-Δ5152 in ducks had disappeared. Those findings suggest that the hypothetical protein, LORF5, UL55, and LORF4 genes of DEV are associated with virus virulence, and the flexibility of this region provided excellent insertion sites for exogenous genes when DEV is used as a recombinant vaccine vector.

## 1. Introduction

Duck virus enteritis (DVE), or duck plague, is a highly contagious and often fatal disease of waterfowl caused by the duck enteritis virus (DEV). Migratory waterfowl enable the virus’s transcontinental spread, leading to its global distribution. Infected ducks shed virus long-term via feces and oral secretions, thereby contaminating the environment with DEV [1]. DEV exhibits broad tissue tropism with systemic dissemination to all major organs post-infection, inducing severe lytic damage. The virus establishes latency reservoirs in trigeminal ganglia, peripheral blood lymphocytes, and lymphoid tissues, which enables the virus to persist long-term within the host and continuously shed into the environment. These characteristics contribute to the rapid transmission, high morbidity, and significant mortality of DEV, establishing it as a severe pathogenic threat to the global waterfowl industry [2,3,4].

Duck enteritis virus (DEV), taxonomically known as *Anatid herpesvirus 1*, is a member of the genus *Mardivirus*, subfamily *Alphaherpesvirinae*, and family Herpesviridae [5,6]. It possesses a linear double-stranded DNA genome of approximately 158 kb, which contains 78 predicted open reading frames (ORFs). The genome is organized into unique long (UL) and unique short (US) regions, flanked by internal and terminal repeat short (IRS/TRS) regions, conforming to the characteristic UL-IRS-US-TRS architecture of D-type herpesviruses [7,8]. To date, the complete genomes of several DEV strains have been sequenced. These include the Chinese commercial vaccine strain VAC (first reported in 2009) [5]; the virulent wild-type strain 2085 (isolated during a 2005 outbreak in Germany) [9]; and the highly pathogenic Chinese strain CHV (published in 2012) [10]. The large genomic capacity of DEV, characteristic of herpesviruses, enables its development as a live viral vector by allowing for the insertion and expression of foreign antigens, making it a favorable candidate for a live viral vector [11,12]. Nevertheless, due to the complexity of the DEV genome, the currently available data are insufficient for a comprehensive understanding of the virus [13].

DEV establishes latency in the trigeminal ganglia and lymphoid tissues, while inducing long-lasting humoral immune responses. This capability has established DEV as a critical tool in the development of novel avian vaccines. The recombinant DEV vector expressing the F protein of Newcastle disease virus (NDV) provided 100% protection against lethal NDV infection in chickens [12]. The recombinant DEV vector expressing structural proteins N and S1 of infectious bronchitis virus (IBV) conferred complete (100%) protection against virulent IBV challenge [14]. The recombinant H5N1 avian influenza virus HA protein expressed by duck enteritis virus (DEV) can provide rapid protection against both DEV and H5N1 virus infection in ducks [15].

To investigate DEV pathogenesis, we generated an attenuated strain, E74, by serial passaging of the virulent E1 strain. E74 was non-pathogenic and elicited a rapid, high-titer antibody response in ducks. Genomic analysis revealed major deletions in E74, including a 5.2-kb segment in the UL region that was critically responsible for attenuation. These results establish E74 as a promising and safe vaccine candidate for further development.

## 2. Materials and Methods

### 2.1. Cells, Virusess and Plasmids

The virulent DEV strain E1 and attenuated DEV strain E74 were acquired from the Shanghai Veterinary Research Institute Collection and propagated in primary chicken embryo fibroblasts (CEFs). SPF shelduck were purchased from the National Laboratory Animal Resources Bank of Poultry. The recombinant DEVs were propagated in CEFs, which were prepared from 9-day-old embryonating SPF chicken eggs and maintained in DMEM medium (Hyclone, Logan, UT, USA) supplemented with 10% fetal bovine serum (FBS; Biowest, South American origin, Riverside, MO, USA), 100 U/mL penicillin, and 100 μg/mL streptomycin (Sangon Biotech (Shanghai) Co., Ltd., Shanghai, China) at 37 °C, with 5% CO_2_. Infectious supernatants were harvested when cytopathic effect (CPE) reached >80%, clarified by low-speed centrifugation (2000× *g*, 10 min), aliquoted, and stored at −80 °C. Whole-genome sequencing of the virus was performed on an Illumina MiSeq platform by GENEWIZ (Suzhou, China).

The sgRNAs were designed according to https://benchling.com (accessed on 15 July 2025) and targeted the LORF5-, UL55-and LORF4-encoding open reading frames (ORFs). The sequences of the sgRNAs are listed in Table 1. The PX330 plasmid was digested with BbsI restriction enzyme and subsequently ligated with the annealed sgRNA product.

### 2.2. Viral Growth Kinetics in CEF Cells

To determine viral growth kinetics, CEF cells were infected with each virus at a multiplicity of infection (MOI) of 0.001. The cells were incubated at 37 °C for 2 h, the cells were washed 3 times with PBS and further incubated with DMEM containing 2% FBS at 37 °C, 5% CO_2_. The cell supernatants were collected at 0, 24, 48 and 72 dpi and titrated by inoculating CEF cells in 96-well plates. Three independent experiments were performed. The TCID_50_ value was calculated by the Reed–Muench method.

### 2.3. Generation of Recombinant Viruses

The recombinant virus rE1-Δ5152 was generated using a CRISPR/Cas9-assisted method. Primary CEF monolayers were co-transfected with a mixture of three sgRNA plasmids (PX330-sgRNA-LORF5, -sgRNA-UL55, and -sgRNA-LORF4; 1 µg total) using Lipofectamine 3000 (Thermo Fisher Scientific, Waltham, MA, USA). At 8 h post-transfection, the cells were infected with the parental E1 strain at a multiplicity of infection (MOI) of 0.1. The cultures were incubated at 37 °C with 5% CO_2_ until extensive CPE was observed (approximately 90% cell detachment). The infected cells were then harvested, subjected to three freeze–thaw cycles, and the clarified supernatant was used for three sequential rounds of plaque purification on fresh CEFs to isolate the pure recombinant virus, rE1-Δ5152.

### 2.4. The Pathogenicity Experiment in Ducks

To evaluate the comparative pathogenicity of E1 and E74 strains in ducks, each experimental group comprised six 8-day-old SPF Shelducks were inoculated intramuscularly (i.m.) with 10^5.0^ TCID_50_ of each virus at a volume of 0.2 mL, respectively. The control group was i.m. injected with 0.2 mL of PBS. In each group, three inoculated ducks were euthanized at 4 dpi by CO_2_ inhalation, and the tissue samples of heart, liver, spleen, lung, kidney, duodenum, pancreas and brain were collected for viral titration. Liver and spleen samples will be collected for histopathological analysis. The remaining three ducks in each group were monitored for 14 days to record survival. Serum samples obtained at 0 and 7 dpi were used for antibody level quantification.

To further investigate the impact of the genetic deletion on pathogenicity, the pathogenicity of the recombinant rE1-Δ5152 was tested against the virulent strain E1. Groups of ducks (*n* = 6) were inoculated i.m. with 10^5.0^ TCID_50_ of either virus. Following the same protocol, three ducks per group were sacrificed at 4 dpi for extensive tissue sampling (heart, liver, spleen, lung, kidney, duodenum, pancreas, brain) to determine viral loads and pathological changes. The remaining ducks were observed for survival for 14 days.

### 2.5. Virus Titration

The viral load in tissues was quantified via a TCID_50_ assay in CEF cells. The tissue homogenates were clarified by centrifugation at 2000× *g* for 10 min at 4 °C. Supernatants were collected and subjected to serial 10-fold dilutions in DMEM. Confluent monolayers of CEF cells in 96-well plates were inoculated with 100 μL of each dilution. Following 1 h adsorption at 37 °C with 5% CO_2_, the inoculum was replaced with DMEM containing 2% FBS. Cultures were observed for CPE over 6 days. Viral titers (log_10_ TCID_50_/0.1 g tissue), calculated by Reed–Muench method, with a defined assay sensitivity of 0.5 log_10_ TCID_50_/0.1 g of tissue.

### 2.6. Blocking ELISA

Anti-DEV antibodies in serum samples were quantified using the DEV antibody detection method previously established in the laboratory. Serum samples were diluted 1:10 in sample dilution buffer and added to antigen-coated wells (100 μL/well) in duplicate. Following 1 h incubation at 37 °C, plates were washed three times with PBST. Mouse anti-DEV monoclonal antibody was added as the primary antibody and incubated for 1 h at 37 °C. After washing, horseradish peroxidase (HRP)-conjugated goat anti-mouse IgG (1:2000; Sigma, St. Louis, MO, USA) was added and incubated for 1 h at room temperature. After incubating for 1 h at room temperature and washing with PBST 3 times, 3,3′,5,5′-tetramethyl benzidine was added, and the mixture was incubated at room temperature for 8 min. The reaction was then stopped by adding 0.1 N sulfuric acid. The optical density (OD_450nm_) was measured, and the percent inhibition (PI) was determined using the following formula: PI (%) = [1 − (OD_450_ of test serum/OD_450_ of negative-control serum)] × 100%. The serum was considered positive for DEV reactivity when the PI value was ≥21.6%.

### 2.7. Statistical Analysis

All animal experiments were carried out in accordance with the recommendations in the Guide for the Care and Use of Laboratory Animals of the Ministry of Science and Technology of China. The protocol (SV-20231013-Y04) used in the study was approved by the Animal Care Committee of the Shanghai Veterinary Research Institute. All statistical analyses were performed with GraphPad Prism version 7.0 (GraphPad Software, Inc., San Diego, CA, USA). Data are expressed as means with standard deviations (SD). The differences are considered significant at *p* < 0.05 and extremely significant at *p* < 0.01.

## 3. Results

### 3.1. Growth Kinetics of E1 and E74 in CEF Cells

**Figure 1:**
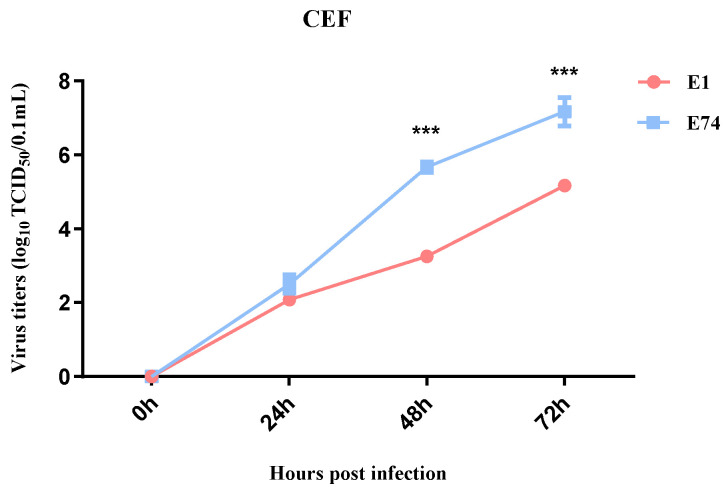
Viral growth kinetics. CEF was infected with E74 or E1 (MOI = 0.001), the supernatants were collected at the indicated time points for virus titration in CEF cells. The data for virus titers indicate the means of the results of three repeats, and the error bars indicate standard errors of the means (***, *p* < 0.001).

To compare the replication capacity of the attenuated strain E74 with the wild-type strain E1 in CEF cells, growth kinetics of E74 and E1 were determined in CEF cells. Both viruses were inoculated at an MOI of 0.001 and measured virus titers in the supernatants at different time points post infection. No significant difference in viral titer was observed between the two strains at 24 hpi. However, at 48 and 72 hpi, the E74 strain yielded a roughly 100-fold higher titer than the E1 strain (Figure 1). These results demonstrate that serial passaging rendered E74 adaptation to CEF cells with enhanced replication efficiency.

### 3.2. Pathogenicity Analysis of E1 and E74

**Figure 2:**
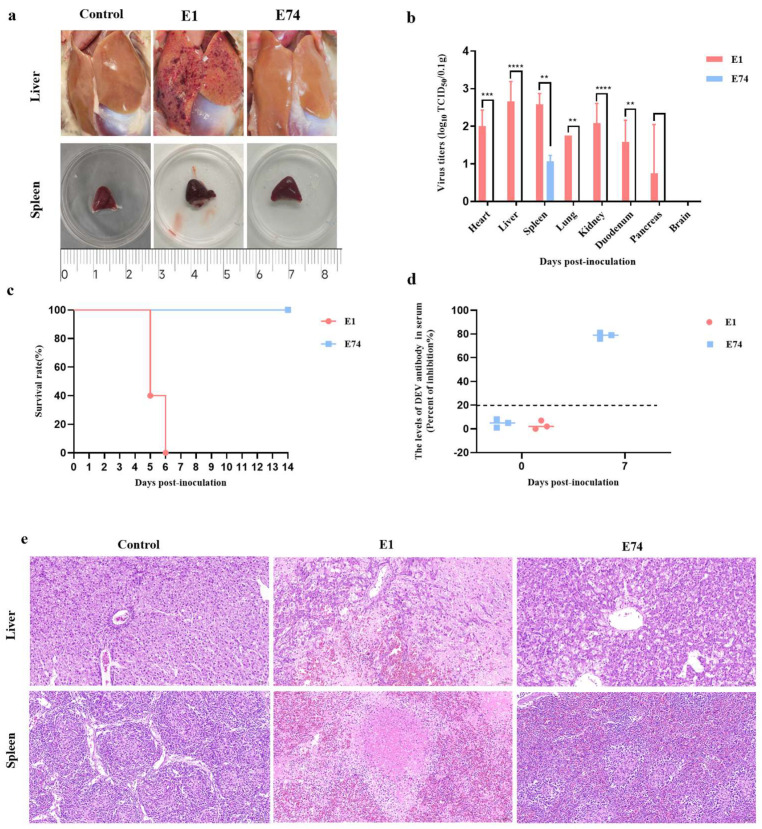
The Pathogenicity of the E74 and E1 in Ducks. Eight 8−day−old Shelducks were inoculated i.m. with 10^5.0^ TCID_50_ of E74 or E1. (**a**) The lesions of livers and spleens of ducks inoculated with the E1 and E74 at 4 dpi. (**b**) The virus titers in all three ducks sampled at 4 dpi. The data for the virus titers indicate the means of the results of three ducks, and the error bars indicate standard errors of the means (**, *p* < 0.01; ***, *p* < 0.001; ****, *p* < 0.0001). (**c**) The survival rates of ducks were monitored daily for 14 consecutive days. (**d**) Serum antibody responses against DEVs were detected at 0 and 7 dpi. Serum was considered positive when the PI value was ≥21.6%. (**e**) Histopathological examination (H&E staining) in the livers and spleens of ducks inoculated with the E74 and E1. Magnification: 20×.

To comparatively assess the virulence of E1 and E74 in ducks, groups of 8-day-old SPF Shelducks were i.m. inoculated with 10^5.0^ TCID_50_ of each virus and monitored for 14 dpi. At 4 dpi, three ducks from each group were euthanized for pathological examination and viral titration. Pathological analysis revealed that all E1-inoculated ducks exhibited marked multifocal hemorrhagic foci on the hepatic surface, accompanied by significant splenomegaly. In contrast, ducks infected with E74, similar to the PBS-inoculated controls, exhibited no notable hepatic lesions (Figure 2a). Viral titration assays further demonstrated that E1 infection resulted in broad tissue tropism, with high viral loads detected in the heart, liver, spleen, lungs, kidneys, and duodenum. Among these organs, the liver and spleen displayed significantly higher viral titers. Only one duck showed detectable virus in the pancreas, while all three ducks remained negative for viral detection in brain tissues. Conversely, the E74 virus can only be detected at a relatively low viral load in the spleen (Figure 2b).

A survival analysis was conducted to evaluate the lethality of the two viruses by observing three ducks per group for 14 days. The data demonstrated that there was no mortality in the E74-infected group, whereas E1 inoculation resulted in 60% lethality at 5 dpi, reaching 100% mortality at 6 dpi (Figure 2c). To evaluate the humoral immune response elicited by the E74 strain, we measured the levels of anti-DEV antibodies in serum samples collected at 0 and 7 dpi using a blocking ELISA. A robust antibody response was detected as early as 7 dpi (Figure 2d). This rapid immunogenicity suggests that E74 is a promising candidate for a live-attenuated vaccine.

H&E staining was performed on liver and spleen tissues exhibiting the highest viral loads. Histopathological analysis revealed that in ducks of the E1 infection group, the liver displayed extensive focal necrosis, along with hepatocellular edema and vacuolar degeneration. Enhanced cytoplasmic eosinophilia was observed, accompanied by mild infiltration of lymphocytes and macrophages. Splenic tissues exhibited extensive necrotic foci, dissolution of white pulp architecture, and widespread congestion of the red pulp. In contrast, ducks of the E74 infection group showed no significant histopathological abnormalities in either liver or spleen tissues (Figure 2e).

### 3.3. Comparative Genomic Analysis of E1 and E74 and Rescue of a rE1-Δ5152 Recombinant Virus

**Figure 3:**
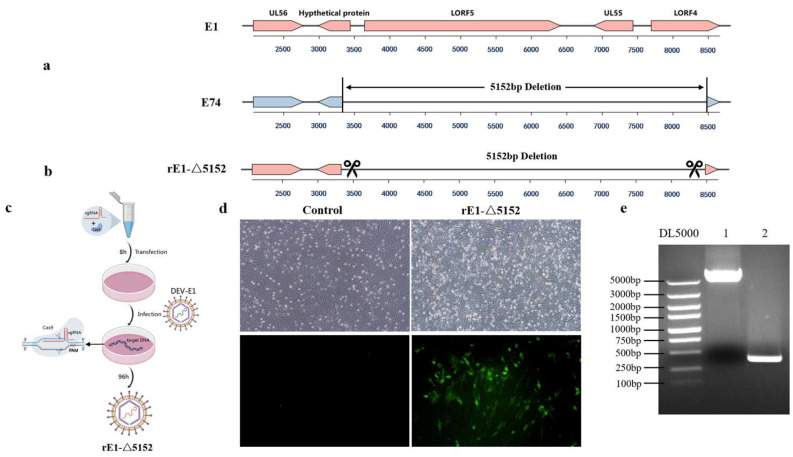
The comparison of genomic organization between the E1 strain and the E74 strain. (**a**) The E74 strain exhibits a gene deletion in the UL region. (**b**) The recombinant viral strain rE1-Δ5152 was generated by deleting a 5152-bp fragment in the UL region of the E1 strain. (**c**) Schematic diagram of recombinant virus rescue. (**d**) Detection via IFA with anti-DEV monoclonal antibody at 48 hpi after rE1-Δ5152 recombinant virus infection in CEF cells. (**e**) A 5152-bp deletion in the UL region of the recombinant virus was verified by PCR.

Comparative genomic analysis revealed distinct large-scale deletions in the attenuated strain E74 relative to the virulent E1 strain. The E74 strain displays a more extensive 5152-bp deletion in the UL region, comprising a 111 bp deletion from the initiation codon of the hypothetical protein gene, a 2772 bp deletion in the LORF5 gene, a 561 bp deletion in the UL55 gene, and a 785 bp deletion starting from the initiation codon of the LORF4 gene (Figure 3a).

To further investigate whether the deletion of these genes attenuates DEV in ducks, a rescued virus rE1-Δ5152 with the 5152 bp deletion in the UL region was generated on the E1 backbone (Figure 3b), and the recombinant virus (designated rE1-Δ5152) was successfully rescued (Figure 3c). Infection of CEF cells with this recombinant virus induced distinct cytopathic effects (CPEs). Monoclonal antibody-based immunofluorescence assay (IFA) against DEV also detected specific fluorescence signals (Figure 3d). PCR amplification followed by sequencing confirmed a 5152-bp deletion at this locus compared to the virulent strain E1, verifying successful rescue of the recombinant virus (Figure 3e).

### 3.4. The Recombinant Virus rE1-Δ5152 Was Attenuated in Ducks

**Figure 4:**
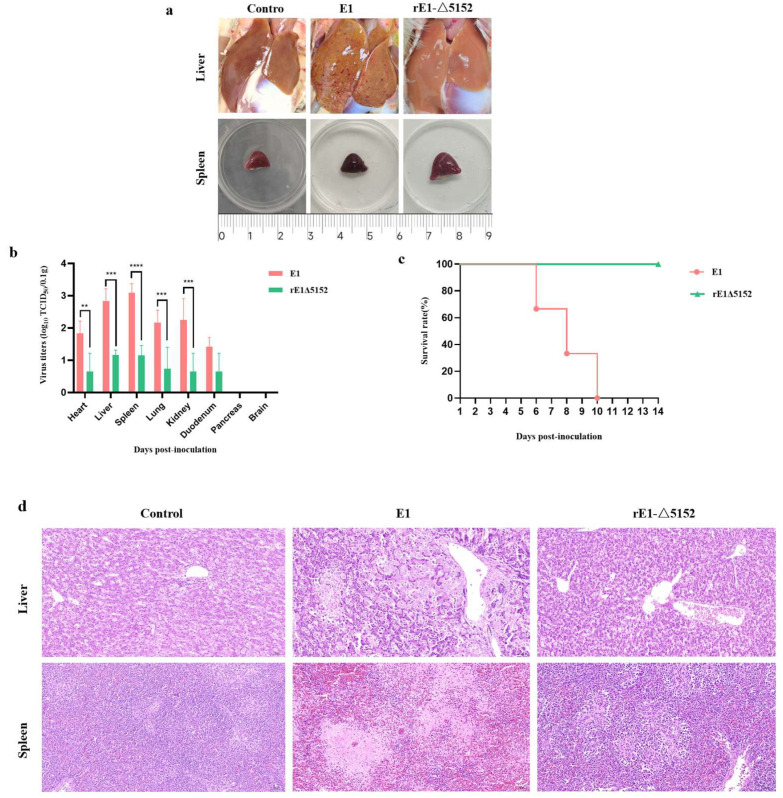
The Pathogenicity of the E1 and rE1-Δ5152 in Ducks. Six 8-day-old Shelducks were inoculated i.m. with 10^5.0^ TCID_50_ of E1 or rE1-Δ5152. (**a**) The lesions of livers and spleens of ducks inoculated with the E1 and rE1-Δ5152 at 4 dpi. (**b**) The virus titers in all three ducks sampled at 4 dpi. The data for the virus titers indicate the means of the results of three ducks, and the error bars indicate standard errors of the means **, *p* < 0.01; ***, *p* < 0.001; ****, *p* < 0.0001). (**c**) The survival rates of ducks were monitored daily for 14 consecutive days. (**d**) Histopathological examination (H&E staining) in the livers and spleens of ducks inoculated with the E1 and rE1-Δ5152. Magnification: 20×.

To further compare the virulence of E1 and the recombinant virus rE1-Δ5152 in ducks, 8-day-old ducks were i.m. inoculated with 10^5.0^ TCID_50_ of each virus and observed for 14 days. At 4 dpi, three ducks per group were euthanized via CO_2_ inhalation. Gross examination revealed severe hepatic lesions, characterized by petechiae and necrotic foci, and hemorrhagic splenomegaly in E1-infected ducks. Conversely, ducks infected with the rE1-Δ5152 strain displayed no hepatic pathology and only mild splenic enlargement in the absence of hemorrhage (Figure 4a). Viral titers in tissue collected from different groups varied. Results showed that although the rE1-Δ5152 virus was detected in all tissues of the infected ducks, its titers were significantly lower than those in the E1-infected group (Figure 4b). The remaining three ducks were monitored for 14 days, which demonstrated that E1 caused 100% mortality rate by 10 dpi (Figure 4c). Conversely, no mortality was observed in any of the three ducks from the rE1-Δ5152-infected group (Figure 4c). Histopathological examination showed that ducks in the E1-infected group exhibited extensive focal necrosis in the liver, with significant hepatocellular edema. Additionally, splenic tissue showed necrosis and extensive vascular congestion. Conversely, no significant pathological changes were observed in the liver or spleen of rE1-Δ5152-infected ducks (Figure 4d). Taken together, these findings indicate that the LORF5, UL55, and LORF4 genes of duck enteritis virus are critical for its full pathogenicity in ducks, and their simultaneous deletion effectively attenuates the virus.

## 4. Discussion

Since its first report in 1923, duck plague remains sporadically prevalent in waterfowl farming regions worldwide [16]. Characterized by rapid transmission dynamics, broad epidemiological distribution, and elevated morbidity and mortality rates, this pathogen persistently jeopardizes the global duck industry, incurring substantial economic losses across production systems [2]. Duck plague is an acute, febrile, and septicemic disease that causes systemic viral dissemination and is characterized by multi-organ hemorrhages [17]. In this study, infection with the isolated highly virulent DEV strain E1 induced significant pathology in ducks, notably presenting as pronounced petechial hemorrhages and whitish necrotic foci in the liver and marked congestion leading to a dark-red discoloration of the spleen. Viral titers were detected in all tissues sampled except the brain, confirming the broad tissue tropism of the strain. Consistent with its virulence, virulent DEV infection leads to unabated viral replication across host tissues culminating in mortality. Whereas the attenuated DEV vaccine strain can persist at a steady level in the host’s immune organs, it is eliminated from peripheral tissues including the heart, lung, intestine, and blood, where no virus can be detected in the later phase of infection [18]. Interestingly, our study revealed that the attenuated DEV strain E74 exhibited a uniquely restricted tissue distribution; it was detected exclusively in the spleen, with no virus found in any other examined tissues. Currently, no targeted antiviral drugs exist for duck plague, with its control largely dependent on vaccination using live-attenuated vaccines, which constitute the primary commercial products available [19]. With advances in molecular virology, recombinant vaccines constructed using the DEV vaccine strain as a live viral vector have been widely applied against various avian diseases [20].

The DEV is commonly employed as a vaccine vector platform by utilizing targeted deletions within virulence-associated genes as insertion sites for heterologous genes [21]. This strategy confers the key advantages of stable attenuation, sustained high-level expression of the foreign antigen, and significantly reduced overall pathogenicity. Among Alphaherpesviruses, the loci most commonly targeted for deletion include replication-nonessential virulence-associated genes and glycoprotein genes with high immunogenicity, such as TK, gC, gG, gE, and gI. Previous research indicates that FHV-1 mutants with concurrent gI/gE/TK deletions attenuated clinical signs, diminished viral shedding, and enhanced neutralizing antibody responses in vaccinated cats [22]. The BoHV-1 mutant with concurrent gG/gE deletions demonstrates attenuated viral virulence while eliciting robust IFN-β production during early infection and maintains the capacity to stimulate protective immunity [23]. Additionally, studies identified that targeted deletion of both gC and gE in DEV exhibits significantly attenuated viral virulence and is non-pathogenic in ducks, and stable genetic characteristics in vitro and in vivo [24]. This study found that in addition to the previously characterized virulence-associated non-essential genes, four other genes—Hypothetical protein, LORF5, UL55, and LORF4—exhibited deletions of varying lengths. Further validation demonstrated that the simultaneous deletion of these four genes in the virulent DEV strain E1 attenuated its lethality in ducks. These results indicate that the Hypothetical protein, LORF5, UL55, and LORF4 genes are associated with the virulence of duck enteritis virus. Evidence suggests that UL55 is the primary activator of the UL13 kinase in HSV-2, with Us10 serving an accessory function, and that UL13-mediated replication and spread are highly dependent on UL55 [25]. The LORF2, LORF3, LORF4, LORF5, and SORF3 genes are unique to avian herpesviruses, where LORF4 and LORF5 have been shown to be non-essential for viral replication and assembly [26]. Consequently, these genes could be targeted for the insertion of foreign sequences as a strategy for viral vector development.

In this study, an attenuated strain of DEV, designated E74, was successfully generated. The pathogenicity of E74 was evaluated and compared with that of the virulent strain E1 in ducks. The results showed that E74 did not induce clinical symptoms or cause mortality, whereas infection with E1 resulted in 100% mortality. Furthermore, only low levels of the E74 virus were detected, exclusively in the spleen. Most importantly, ducks infected with the E74 strain elicited high-titer antibodies as early as 7 days post-infection. Comparative genomic analysis identified a 5152-bp deletion in the UL region of E74. A recombinant virus (rE1-Δ5152) harboring a 5152-bp deletion in the UL region was successfully rescued from the virulent strain E1 backbone. This mutation resulted in a significant attenuation of viral virulence in ducks. This finding indicate that this region can be exploited as a promising site for inserting exogenous genes to engineer attenuated DEV vaccine vectors. Therefore, future studies should focus on identifying the key virulence genes within this deleted region that are responsible for DEV pathogenicity in ducks, thereby further elucidating the molecular mechanisms of disease pathogenesis.

## Figures and Tables

**Table 1:** Primers used in the study.

Target Gene	Forward Primer	Reverse Primer	Size
sgRNA-LORF5-1	CACCGGATGATGATGAAGACTCCGA	AAACCTCGGAGTCTTCATCATCATC	
sgRNA-UL55-1	CACCGTCGGAGGCGAGACTTGTGTA	AAACCTACACAAGTCTCGCCTCCGA	
sgRNA-LORF4-1	CACCGTACGCCAAGAGCCGAAGCTC	CACCGTACGCCAAGAGCCGAAGCTC	
LORF5~LORF4	GTCTACTGGACGTAGTCTTCATAA	TCACTCATCCGAAGAGTTACACGCA	5536 bp

## Data Availability

The original contributions presented in this study are included in the article. Further inquiries can be directed to the corresponding authors.
